# A Microfluidic Micropipette Aspiration Device to Study Single-Cell Mechanics Inspired by the Principle of Wheatstone Bridge

**DOI:** 10.3390/mi10020131

**Published:** 2019-02-16

**Authors:** Yong-Jiang Li, Yu-Nong Yang, Hai-Jun Zhang, Chun-Dong Xue, De-Pei Zeng, Tun Cao, Kai-Rong Qin

**Affiliations:** 1 School of Optoelectronic Engineering and Instrumentation Science, Dalian University of Technology, Dalian 116024, Liaoning, China; yongjiangli@dlut.edu.cn (Y.-J.L.); xuechundong@dlut.edu.cn (C.-D.X.); 2 School of Biomedical Engineering, Dalian University of Technology, Dalian 116024, Liaoning, China; yangyunong@mail.dlut.edu.cn (Y.-N.Y.); haijunzhang@mail.dlut.edu.cn (H.-J.Z.); 1925044995@mail.dlut.edu.cn (D.-P.Z.)

**Keywords:** micropipette aspiration, microfluidics, single-cell mechanics, Wheatstone bridge

## Abstract

The biomechanical properties of single cells show great potential for early disease diagnosis and effective treatments. In this study, a microfluidic device was developed for quantifying the mechanical properties of a single cell. Micropipette aspiration was integrated into a microfluidic device that mimics a classical Wheatstone bridge circuit. This technique allows us not only to effectively alter the flow direction for single-cell trapping, but also to precisely control the pressure exerted on the aspirated cells, analogous to the feature of the Wheatstone bridge that can precisely control bridge voltage and current. By combining the micropipette aspiration technique into the microfluidic device, we can effectively trap the microparticles and Hela cells as well as measure the deformability of cells. The Young’s modulus of Hela cells was evaluated to be 387 ± 77 Pa, which is consistent with previous micropipette aspiration studies. The simplicity, precision, and usability of our device show good potential for biomechanical trials in clinical diagnosis and cell biology research.

## 1. Introduction

The biomechanical properties of single cells serve as critical factors in directing the physiological functions of cells, such as cell growth, proliferation, and migration, which ultimately contribute to pathophysiological progression [1,2,3]. Typically, cancer cells are more deformable than healthy ones, which facilitates their metastatic journey into the blood stream [1]. Cell adhesion results in the mechanical scaffold for cell cortex tension to drive cell sorting during gastrulation [4]. Intrinsic mechanical changes in cell and tissue structure lead to the development of malignancy and metastasis [5]. Not only cell mechanical properties affect cell functions. On the contrary, biological processes also change the cell mechanics. The stiffness increases as cells enter mitosis [6], as tumor cells transit to premalignant stage [7], and when red blood cells are infected with malaria [8,9]. In this context, the characterization of cellular biomechanics could provide novel insight in understanding the development of human diseases such as tumor and cancer, showing a good potential in early disease diagnosis and effective treatments. Therefore, considerable interest have been aroused in determining the biomechanical properties of single cells.

To date, numerous quantitative micromanipulation techniques have been developed to evaluate the mechanical properties of single cells, such as micropipette aspiration (MPA), optical tweezers, magnetic twisting cytometry, and atomic force microscopy [10]. Among these methods, MPA provides a non-invasive, simple, and direct approach to measure cell mechanics at the single-cell level [11]. The classical MPA experiment consists of partial or complete suction of single cells into a glass micropipette using negative pressure. By recording the cell deformation to applied pressure, the intrinsic mechanical properties of individual cells can be determined using various models [12,13,14,15,16]. Yet, the small pressure control and manual trapping of target cells in suspension or attached cells make MPA operation challenging and time-consuming. Continuous evaporation loss results in a drifting baseline of the aspiration pressure, leading to measurement inaccuracy [14,17]. Recently, Shojaei-Baghini et al. [18] reported an automated MPA. Yet, a proportional–integral–derivative (PID) position controller, motorized pressure system, and real-time visual tracking system are necessary. Although the MPA technique is theoretically straightforward, it requires not only specialized equipment to precisely control the small pressure, but also highly delicate manipulation to manually target the cells at the single-cell level [19].

Recent developments in microfabrication and microfluidic techniques can solve the problems discussed above. Microfluidics have advantages in single-cell manipulation and precise mechanical stimuli loading, which are challenging for traditional MPA. Moreover, microfluidic devices are inherently matched with the individual cell in scale. Therefore, microfluidics is an effective technique for single-cell mechanics studies [20,21]. A variety of microfluidic forms and techniques have been developed for single-cell mechanical characterization, including constriction channel [9,22,23], fluid stress [24,25], optical stretcher [26], electro-deformation, and electroporation. A few researchers have also applied the MPA technique to microfluidic devices. Chen et al. [27] combined an impedance analyzer and MPA for the simultaneous mechanical and electrical characterization of single cells. Guo et al. [9,19] demonstrated a microfluidic micropipette aspiration with two-layer microstructure and membrane microvalves, which takes advantage of fluidic circuitry to attenuate exerted pressure on cells within a funnel constriction channel for mechanical characterization. However, these microfluidic devices mentioned above employed either complicated microfluidic structure design or sophisticated peripheral systems for single-cell manipulation and exerting forces. An easy-to-use microfluidic device is thus required to characterize single-cell mechanics.

Herein, a microfluidic device is proposed for quantifying cell mechanics at the single-cell level by combining the micropipette aspiration technique and the Wheatstone bridge principle. The microfluidic analog of the Wheatstone bridge allows effective trapping of single cells and precise control of the suction pressure on aspirated cells. The combination of MPA can quantitatively measure the cell deformability, revealing the advantages of simplicity in implementation, ease of use, and reduction of sample consumption. The simplicity, precision, and usability of our device show its potential for biomechanical trials in clinical diagnosis and cell biology research.

## 2. Materials and Methods

### 2.1. The Principle of the Microfluidic Wheatstone Bridge

**Figure 1:**
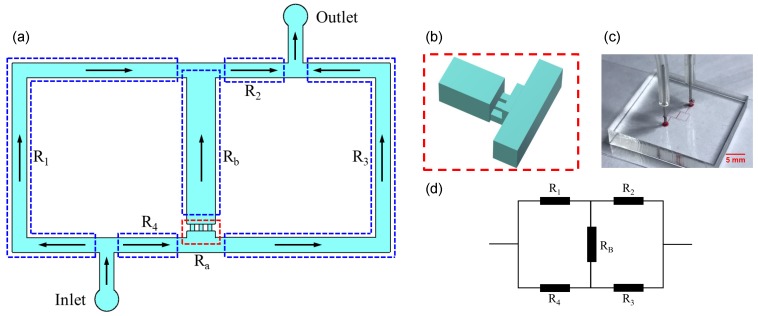
(**a**) Schematic of the Wheatstone bridge microchannel. The microchannel is divided into four segments with distinct flow resistances ($R_{1},R_{2},R_{3},R_{4}$) by the inlet, outlet, and bridge microchannels. (**b**) Micropipette aspiration microchannels. (**c**) A picture of the device. (**d**) The equivalent circuit of the Wheatstone bridge microchannel.

The microfluidic device was designed based on the analog of the classical Wheatstone bridge (Figure 1). We applied the principle to quantitatively regulate the flow rate and pressure difference (equivalent to current and voltage in an electric circuit) through the bridge channel by adjusting flow resistances (Figure 1d).

Following the Darcy–Weisbach equation, the flow resistance of a rectangular microchannel is expressed as [28]:

(1) $$R=\frac{\Delta p}{Q}=\frac{C(\alpha )}{32}\frac{\eta LP^{2}}{A^{3}},$$

where

(2) $$C\left(\alpha \right)=96\left(1-1.3553\alpha +1.9467\alpha^{2}-1.7012\alpha^{3}+0.9564\alpha^{4}-0.2537\alpha^{5}\right),$$

in which α=H/W is the aspect ratio, η is the viscosity, *L* is the microchannel length, Δp is the pressure difference, *Q* is the flow rate, and *P* and *A* are the perimeter and the area of the rectangle cross section, respectively. From Equations (1) and (2), it can be determined that flow resistance only depends on geometry and dimensions for a given solution.

According to the equivalent circuit of the microfluidic Wheatstone bridge (Figure 1d), the flow rate through the bridge channel can be given by:

(3) $$Q_{B}=Q_{t}\frac{R_{1}R_{3}-R_{2}R_{4}}{\left(R_{1}+R_{4}\right)\left(R_{2}+R_{3}\right)+R_{B}\left(R_{1}+R_{2}+R_{3}+R_{4}\right)},$$

where $R_{1}$, $R_{2}$, $R_{3}$, $R_{4}$ are flow resistances, $Q_{t}$ is the total flow rate, and $R_{B}$ is the total resistance of the bridge channel, which is expressed by:

(4) $$R_{B}=R_{b}+R_{a}/N,$$

where $R_{a}$ and $R_{b}$ are the flow resistances of the single aspiration channel and the fractional bridge channel, respectively (Figure 1a). *N* denotes the number of open micropipette aspiration channels (Figure 1b), herein three micropipette aspiration channels were designed. Consequently, the pressure difference of the micropipette aspiration channel can be written as:

(5) $$\Delta p_{A}=\frac{Q_{B}R_{a}}{N}.$$

In the above expressions (Equations (3) and (5)), $Q_{B}$ and $\Delta p_{A}$ are functions of total flow rate and flow resistances, which can be thus quantitatively controlled by regulating $Q_{t}$ and microchannel structures.

To enhance the trapping efficiency, we regulate the flow direction through the bridge channel ($Q_{B}>0$) and ensure its flow rate is larger than that through microchannel $R_{3}$, which should satisfy the conditions:

(6) $$R_{1}R_{3}>R_{2}R_{4}$$

and

(7) $$Q_{B}>Q_{3}=\frac{Q_{B}R_{B}+R_{2}Q_{t}}{R_{2}+R_{3}},$$

where $Q_{3}$ denotes the flow rate within the branch $R_{3}$.

### 2.2. Fabrication and Operation of the Microfluidic Device

The microfluidic MPA device based on the principle of the Wheatstone bridge consists of a polydimethylsiloxane (PDMS)–glass chip fabricated by standard soft-lithography techniques (Figure 1c). The microchannels were patterned in PDMS (Sylgard 184, Dow Corning, Midland, MI, USA) by replica molding. The mold was prepared by spin-coating a thin layer of negative photoresist (SU8-2050, MicroChem, Newton, MA, USA) onto silicon wafers polished on one side (111 N-type, Lijing Ltd., Quzhou, China) and patterned with UV mask aligner (URE-2000/35, Chinese Academy of Sciences, Beijing, China). Next, the micro-channel layer was obtained by pouring PDMS with 10:1 (*w*/*w*) base:crosslinker ratio onto the mold, yielding a thickness of approximately 3 mm. After curing the elastomer for 2 h at 80 ${}^{\circ }$C, the PDMS slab was peeled from the mold, punched, and hermetically bonded to a coverslip by plasma oxidation. In our device, all the microchannels consist of rectangular cross sections. According to the requirements in Equations (6) and (7), the dimensions of microchannels were determined as shown in Table 1.

**Figure 2:**
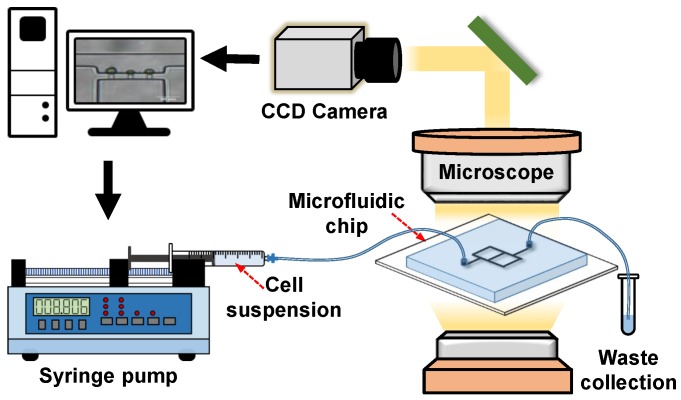
Schematic of the microfluidic micropipette aspiration system.

For the operation of the microfluidic micropipette aspiration device (see Figure 2), the inlet was connected to a syringe pump (Pump 11 Elite, Harvard Apparatus, MA, USA) filled with the cell suspension. During the cell trapping, the cell suspension was pumped at the volume flow rate $Q_{t}$ increased from 20 μL/h to 120 μL/h at a step of 10 μL/h. In each case, we waited at least 1 min to observe the cell deformation. Images of cell deformation were captured only when the cell maintained its shape for at least 1 min (i.e., the stable state). The cell deformation due to the micropipette aspiration was observed under an optical microscope (CKX41, Olympus, Tokyo, Japan) with a CCD camera. The recorded images were further applied to measure the mechanical properties of single cells.

### 2.3. Cell Culture and Sample Preparation

The Hela cell line was purchased from the Cell Resource Center in the Shanghai Institutes for Biological Sciences (SIBS, Shanghai, China). Dulbecco’s Modified Eagle Medium (DMEM/high glucose), fetal bovine serum (FBS), phosphate buffered saline (PBS), trypsin-EDTA, and penicillin/streptomycin were provided by Hyclone (Thermo Scientific, Waltham, MA, USA ). The Hela cell line was cultured in standard culture flasks using DMEM supplemented with 10% FBS, 1% penicillin/streptomycin, and 1% 1-glutamine (Sigma-Aldrich, St. Louis, MO, USA). After the third generation, the cells were collected via trypsin, and then a cell suspension at a density of ∼$10^{6}$ cells/mL was made.

### 2.4. Measurement of Cell Mechanics

**Figure 3:**
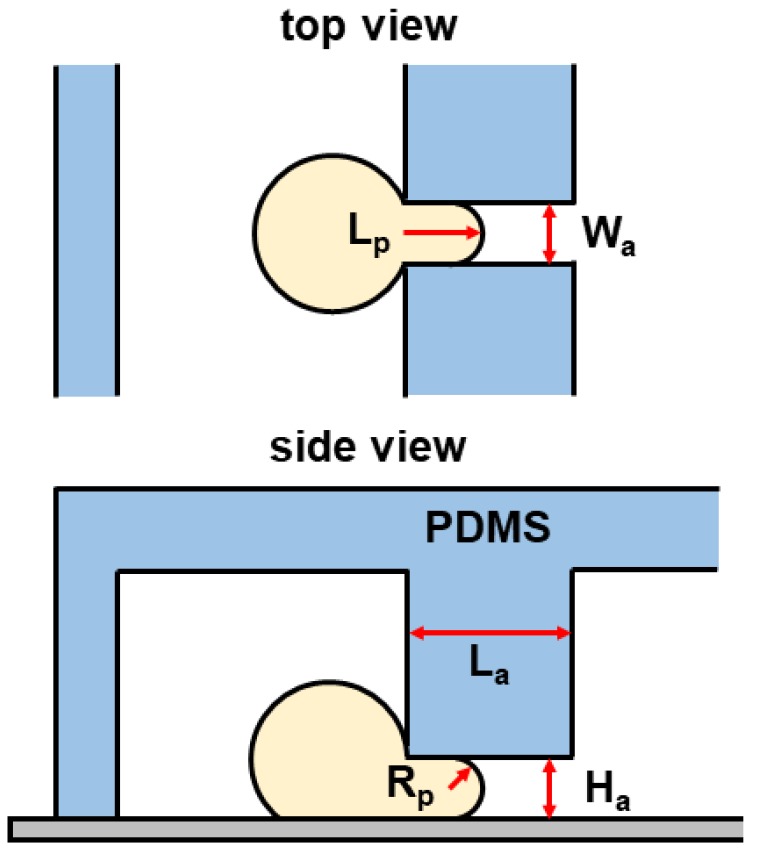
Schematic of a spherical cell aspirated into an micropipette aspiration (MPA) channel with a suction pressure $\Delta p_{A}$. PDMS: polydimethylsiloxane.

In general, the two most popular models for analyzing single-cell mechanics treat the cell either as a homogeneous elastic solid or a drop of liquid encapsulated by an elastic solid shell [14]. Here, we adopt the elastic solid model of Theret et al. [12]. Figure 3 presents a schematic diagram of a spherical cell aspirated into an MPA channel. The Young’s modulus of single cells to pressure is thus expressed as:

(8) $$E=\frac{\frac{3\Delta p_{A}\Phi }{2\pi }}{\left(\frac{L_{p}}{R_{p}}\right)},$$

where *E* is Young’s modulus, $\Delta p_{A}$ is the suction pressure indicated in Equation (5), and Φ is a term that depends on the geometry of the micropipette. A typical value for Φ is Φ=2.1. $L_{p}$ denotes the extension length of the surface of the cell into the micropipette (see Figure 3). $R_{p}$ is the hydraulic radius of the micropipette aspiration channel [29], which can be given as:

(9) $$R_{p}=\frac{W_{a}H_{a}}{W_{a}+H_{a}},$$

where $W_{a}$ and $H_{a}$ are the width and height of the micropipette aspiration channel, respectively (see Figure 3).

In the measurement procedure, we measured the protrusion length $L_{p}$ into the micropipette aspiration channel at the stable state, where no significant deformation occurred for at least 1 min. The suction pressure $\Delta p_{A}$ was calculated with the analytical results in Equation (5) according to the corresponding volume flow rate $Q_{t}$. The Young’s modulus of a Hela cell was then evaluated with Equation (8). All the measurements were calibrated by measuring the bridge channel width (88 μm) in pixels. Note that cell mechanics were only characterized when the cell behaved as a solid for the protrusion length $L_{p}\leq L_{a}/2$ at the stable state. The instances where cells flowed entirely into or passed through the MPA channels were not considered.

### 2.5. COMSOL Simulation

The velocity and pressure fields were numerically studied using COMSOL Multiphysics. A 3-D simulation was conducted with the dimensions indicated in Table 1. Using the linear flow module (spf), the velocity and pressure distributions were measured at the flow rate $Q_{t}$ increased from 20 μL/h to 120 μL/h at the step of 20 μL/h. The simulation results of aspiration pressure $\Delta p_{A}$ were calculated by averaging the pressure drops along the centerline of the micropipette aspiration channels. In addition, the particle tracing module (fpt) was applied to track the microparticle movements within the microchannel, which was used to evaluate the trapping efficiency of the micropipette channels.

## 3. Results

### 3.1. Quantitative Control of Aspiration Pressure

**Figure 4:**
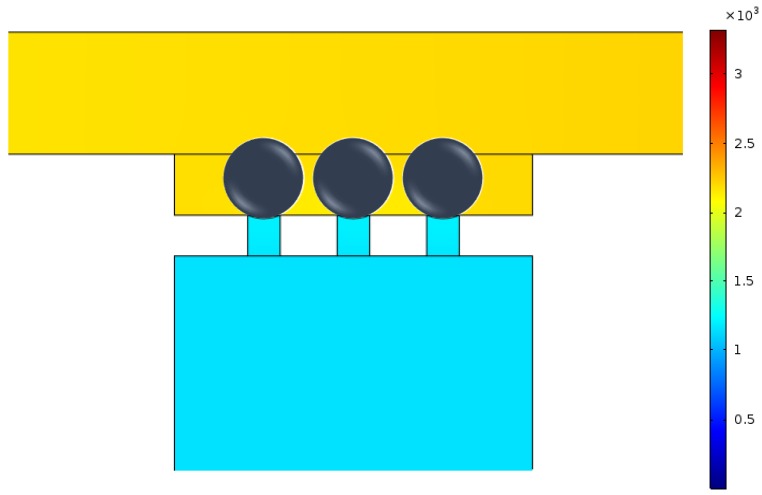
Pressure distribution around the micropipette aspiration channels.

**Figure 5:**
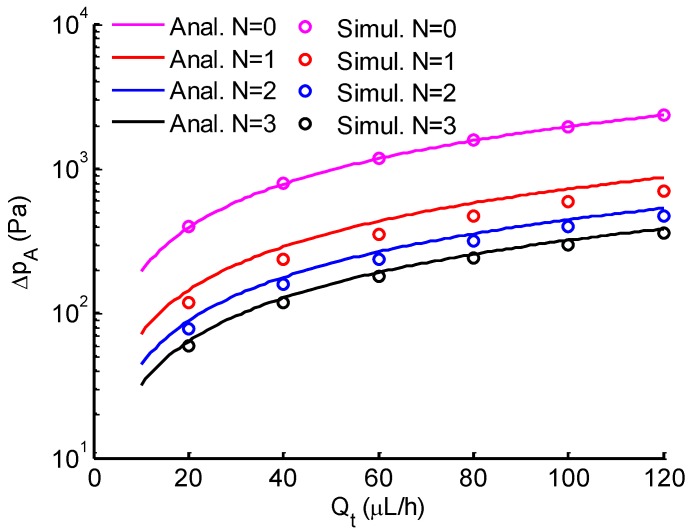
Comparison between analytical (solid lines) and simulation (circle markers) results of the pressure difference across the micropipette aspiration channel versus the total flow rate. *N* is the number of open aspiration channels.

Micropipette aspiration relies on the suction pressure exerted on a single cell to study its biomechanical properties. Firstly, the pressure difference exerted on trapped cells was investigated both analytically and numerically. When single cells were trapped by the micropipette aspiration channels (N=0), it showed a uniform pressure field at both ends of the micropipette aspiration channels (see Figure 4). When *N* (N=1,2, or 3) micropipette aspiration channels were open, the pressure decreased linearly along the centerline of the open micropipette aspiration channel, in which the pressure difference between its two ends was measured at different flow rates $Q_{t}$. The results were compared with analytical ones calculated with Equation (5). The pressure difference was linearly proportional to the volume flow rate $Q_{t}$ (Figure 5). These two results showed a discrepancy of 10% at a maximum flow rate ($Q_{t}=120$ μL/h), revealing that Equation (5) is reliable for the calculation of the pressure difference across the micropipette aspiration channel.

### 3.2. Effective Trapping of Microparticles and Single Cells

**Figure 6:**
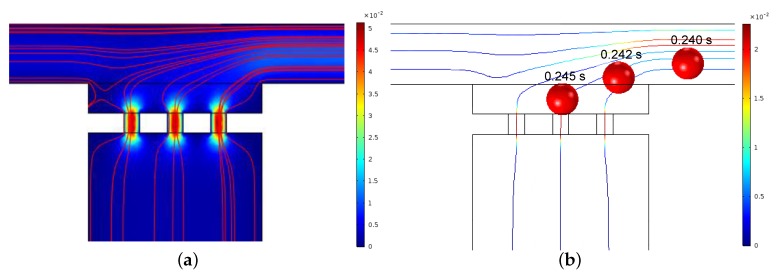
(**a**) Velocity distribution and streamlines around the micropipette aspiration channels for *N* = 3. (**b**) Tracking trajectory of a particle at different time intervals.

**Figure 7:**
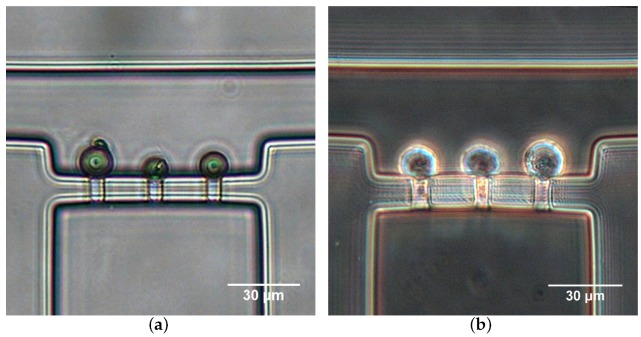
Microparticle (**a**) and Hela cell (**b**) trapping with micropipette aspiration channels at $Q_{t}=40$ μL/h.

The hydrodynamic trapping efficiency of the micropipette aspiration channels was validated both numerically and experimentally. Figure 6a illustrates the numerical simulation result of velocity distribution and streamlines in the region of micropipette aspiration channels. When a microparticle suspension was introduced into the inlet at a velocity of 0.01 m/s, the microparticle close to the side wall flowed along the streamlines and ultimately entered a micropipette aspiration channel (Figure 6b). The trapping efficiency was validated experimentally by introducing microparticle and cell suspension. In both cases, either microparticles or single cells were feasibly trapped by the micropipette aspiration channels (Figure 7), revealing the trapping/aspiration effectiveness of the microfluidic device. In particular, the same cell population showed a different mechanical property, indicated by the variations in protrusion length into the aspiration channels (Figure 7b).

### 3.3. Biomechanical Properties of Single Cells

**Figure 8:**
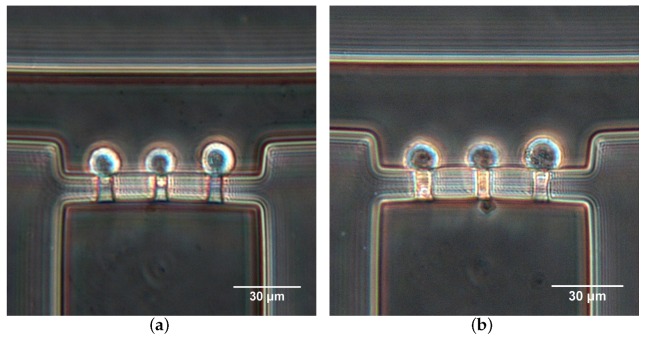
Hela cells aspirated into the micropipette aspiration channels at (**a**) $Q_{t}=40$ μL/h and (**b**) $Q_{t}=30$ μL/h.

Figure 8 shows a demonstration of Hela cells aspirated into the micropipette aspiration channels. To the same applied suction pressures, cells presented different changes in shape, revealing the heterogeneous mechanical properties of cell populations (Figure 8). Additionally, the cell deformation mainly included two forms. In one case, cells showed hemispherical projections into the MPA channels (Figure 8a). In another case, cell membranes extended completely into or even passed through the MPA channels. The two cases respectively demonstrate the solid-like and liquid-like behaviors of cells.

**Figure 9:**
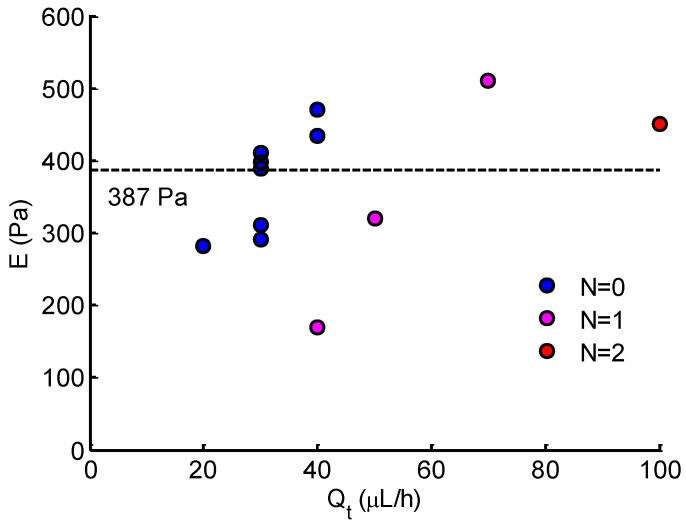
Young’s modulus of Hela cells calculated at different $Q_{t}$. *N* denotes the number of open micropipette aspiration channels.

The Young’s modulus of Hela cells was evaluated for solid-like cells at the stable state. The results are shown in Figure 9. The average Young’s modulus of Hela cells was 387 ± 77 Pa. This value is comparable to previous studies using magnetic tweezer [30] or micropipette aspiration technique [31], but it differs with that measured by atomic force microscopy [3,32,33,34,35]. It is clear that most measurements were at low flow rate $Q_{t}$ (20–40 μL/h), where the aspiration pressure is low. As $Q_{t}$ increased, only four measurements included one or two open micropipette channels (N=1 or N=2), indicating a relatively higher Young’s modulus than those at low flow rates.

## 4. Discussion

The micropipette aspiration technique has been widely used in recent cell biology research, such as cell mechanical properties [19,29], molecular mechanics [17], cell response to mechanic stimuli, and single cell manipulation. Conventional MPA involves delicate manipulations conducted with specialized equipment by highly skilled technicians. Significant evaporation in the chamber leads to a drift in the null setting for the pressure [14]. Few studies have applied a microfluidic platform to conduct MPA for single-cell mechanics characterization, yet complicated microfluidic structures and sophisticated peripheral systems are required for implementation [19,27,36]. In this study, we presented a novel and easy-to-use microfluidic device by coupling the MPA technique and the principle of the Wheatstone bridge circuit. Typically, a classical Wheatstone bridge is an electrical circuit used to measure an unknown electrical resistance by balancing two legs of a bridge circuit, one leg of which includes a variable resistance. It reveals the advantages of high measurement accuracy and simple operation. Owing to these advantages and the comparability between the electrical field and flow field, the principle of the Wheatstone bridge circuit has been employed to control the flow in microfluidic devices [37,38]. Although a microfluidic Wheatstone bridge allows control of the bridge pressure and flow direction by a variable fluidic resistance, it causes difficulties in fabrication and problems in quantifying the resistance through the membrane deformation [38], leading to a quantitative control of the bridge pressure. In order to simplify the problem, in this study, we used fixed resistances and regulated the bridge pressure by controlling the flow rate. The designed device enables effective alteration of the flow direction for single-cell trapping and precise control of suction pressure in the micropipette aspiration channels. The combination of the MPA technique in the microfluidic Wheatstone bridge further improved the trapping efficiency (Figure 7) and provided a quantitative measurement of cell deformability (Figure 8). After image capture, by withdrawing the cell suspension at the inlet, aspirated cells can be released for further cell mechanics measurements. Thus, this device can be used for the long-term study of single cells’ mechanical analysis.

The intrinsic mechanical properties of single cells is closely related to cell adhesion, migration, and motility [1,2,3]. Typically, Young’s modulus is regarded as a biomarker of cell motility, especially in estimating the metastasis of cancer cells. Based on the distinctions between healthy and diseased cells [3,35], Young’s modulus is suggested to be a diagnostic marker in the clinical setting. For the MPA technique, Young’s modulus is determined by measuring deformation to applied force in conjunction with theoretical models [12,13,14,15,16]. The homogeneous half-space model [12,17] applied in this study is based on the assumption of small deformation. The model is employed best to problems in which the displacements and velocities are small. From the Young’s modulus results in Figure 9, most measurements were conducted at low flow rate $Q_{t}$ (20–40 μL/h) due to the small deformations. As the flow rate increased (corresponding to high pressure), the protrusion edge reached or extended the length of the micropipette channels (see Figure 8b). In these cases, the deformation was too large, such that the calculated Young’s modulus may have been underestimated. From this viewpoint, the measurements were conducted only when the protrusion length was less than half of the micropipette channel length at the stable state. This is why only three measurements were included for high flow rate ($Q_{t}>$ 40 μL/h). Note that the analytical aspiration pressure (see Figure 5) was calculated with the average dimensions indicated in Table 1, which was further applied to evaluate Young’s modulus. In fact, due to the fabrication error, the actual dimensions were slightly different from the set values, which may have had a slight impact on measurement accuracy.

In Figure 9, it is shown that the measurement number markedly declined at a high flow rate $Q_{t}$. The measurement could only be obtained in the case of 1 or 2 open micropipette channels (N=1 or N=2). According to the analytical results of pressure (see Figure 5), the increase in the number of open micropipette channels resulted in the decrease in suction pressure. From this point, more open micropipette channels (N≥1) will benefit the measurement at a high flow rate, corresponding to high pressure. Thus, to improve the device and make it work effectively in a wide force range, properly increasing the number of micropipette channels may extend the measurement to a high applied force. In addition, an alternative enabling the device to be available for high force is to improve or develop a theoretical model for large deformations to applied force.

The discussion above reveals that the present microfluidic device combines the advantages of the MPA technique and the Wheatstone bridge principle, which shows the potential for the biomechanical characterization of single cells. This technique also has limitations. One is the realization of high throughput. Although the device can trap and release cells for repetitive and long-term study, repetitive operations lead to inconvenience and changes in cell mechanics. Future improvements will focus on improving the high-throughput capacity of the device by optimizing the structures (e.g., distributing the designed chip in a starlike disposition with the same cell suspension inlet). In this way, more measurements could be achieved simultaneously.

## 5. Conclusions

In this study, we developed a novel microfluidic device for quantifying cell mechanics at the single-cell level. The designed device combines the micropipette aspiration technique and the Wheatstone bridge principle, which allows single cells to be trapped effectively, precise control of the suction pressure, and quantitative measurement of the deformability, revealing the advantages of simplicity in implementation, ease-of-use, and reduction of sample consumption. The simplicity, precision, and usability of our device show great potential for biomechanical trials in clinical diagnosis and cell biology research.

## Figures and Tables

**Table 1:** Dimensions and resistances of microchannels in the microfluidic Wheatstone bridge. Microchannels are denoted by their flow resistances.

Microchannel	Length (μm)	Width (μm)	Height (μm)	Resistance (×10 ${}^{14}$ N·s/m${}^{5}$)
$R_{1}$	5000	30	30	1.76
$R_{2}$	1000	30	30	0.35
$R_{3}$	5000	30	30	1.76
$R_{4}$	1000	30	30	0.35
$R_{b}$	1990	88	30	0.13
$R_{a}$	10	8	8	0.69
